# Supplementary material for: Clinical, Pathological and Prognostic Features of Rare BRAF Mutations in Metastatic Colorectal Cancer (mCRC): A Bi-Institutional Retrospective Analysis (REBUS Study)
Source: Cancers (Basel). 2021 Apr 27;13(9):2098. doi: 10.3390/cancers13092098 (PMC8123617; doi:10.3390/cancers13092098)
Supplement: Supplementary file 1 [file cancers-13-02098-s001.zip › cancers-1137128-supplementary.pdf]

*cancers*

MDPI

# Clinical, Pathological and Prognostic Features of Rare BRAF Mutations in Metastatic Colorectal Cancer (mCRC): A Bi-Institutional Retrospective Analysis (REBUS Study)

**Maria Alessandra Calegari, Lisa Salvatore, Brunella Di Stefano, Michele Basso, Armando Orlandi, Alessandra Boccaccino, Fiorella Lombardo, Alessandra Auriemma, Ina Valeria Zurlo, Maria Bensi, Floriana Camarda, Marta Ribelli, Raffaella Vivolo, Alessandra Cocomazzi, Carmelo Pozzo, Michele Milella, Maurizio Martini, Emilio Bria and Giampaolo Tortora**

| | mOS (months) | HR | 95%CI | *p* |
|---|---|---|---|---|
| *BRAF* V600E | 20.4 | 1.91 | 1.1–3.29 | 0.002 |
| *RAS/BRAF* wild type | 36.1 | | | |

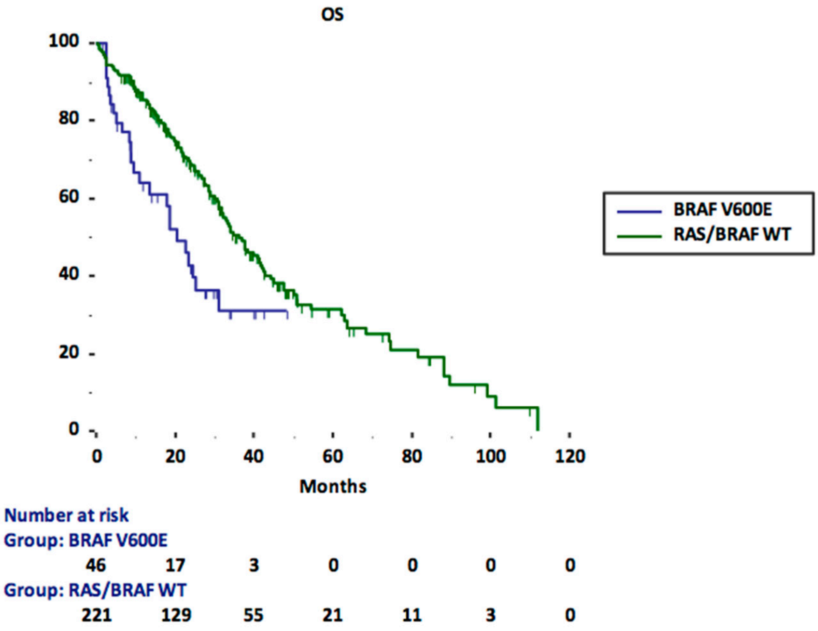


Figure S1: Kaplan-Meier OS curves for *BRAF* V600E cohort vs *RAS/BRAF* wild type dataset

| | mOS (months) | HR | 95%CI | *p* |
|---|---|---|---|---|
| ***BRAF*** **V600E** | 20.4 | 1.59 | 0.96–2.64 | 0.023 |
| ***RAS*** **MT** | 30.3 | | | |

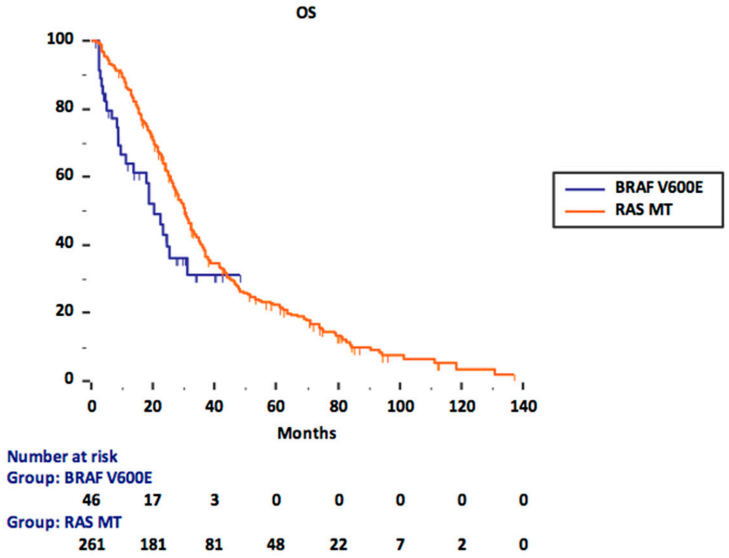


Figure S2: Kaplan-Meier OS curves for *BRAF* V600E cohort vs *RAS* mutated dataset

| | mOS (months) | HR | 95%CI | *p* |
|---|---|---|---|---|
| ***BRAF*** **non-V600** | 61.3 | 0.67 | 0.29–1.54 | 0.43 |
| ***RAS/BRAF*** **wild type** | 36.1 | | | |

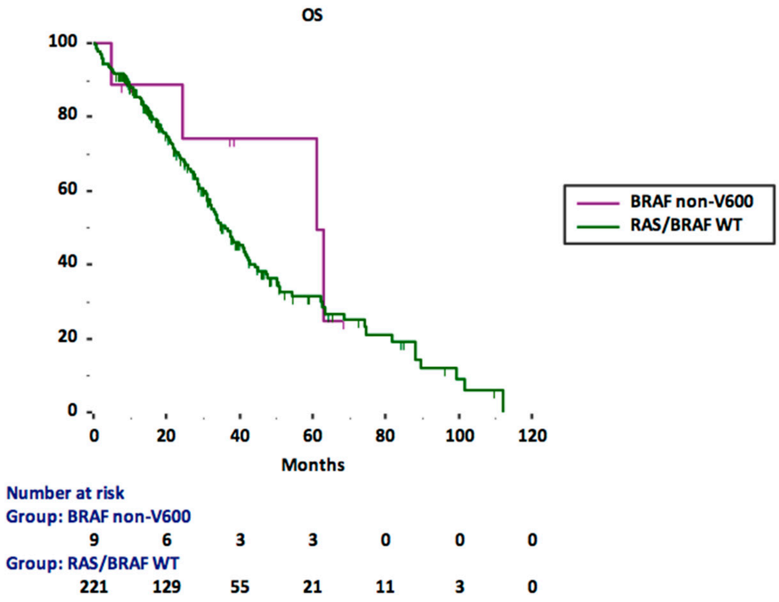


Figure S3: Kaplan-Meier OS curves for *BRAF* nonV600 cohort vs *RAS* mutated dataset

| | mOS (months) | HR | 95%CI | *p* |
|---|---|---|---|---|
| ***BRAF*** **non-V600** | 61.3 | 0.53 | 0.25–1.11 | 0.2 |
| ***RAS*** **MT** | 30.3 | | | |

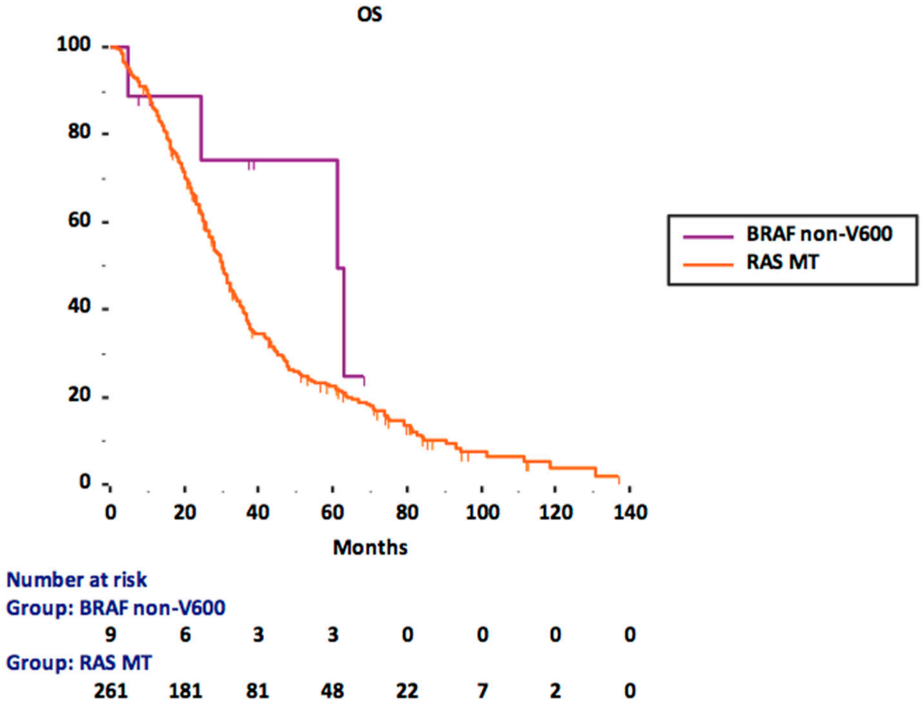


Figure S4: Kaplan-Meier OS curves for *BRAF* nonV600 cohort vs *RAS*/*BRAF* wild type dataset
